# S58. A RANDOMIZED CONTROLLED TRIAL COMPARING VIRTUAL REALITY THERAPY TO COGNITIVE BEHAVIORAL THERAPY IN SCHIZOPHRENIA WITH TREATMENT REFRACTORY HALLUCINATIONS: PRELIMINARY RESULTS

**DOI:** 10.1093/schbul/sby018.845

**Published:** 2018-04-01

**Authors:** Laura Dellazizzo, Stéphane Potvin, Kieron O’Connor, Alexandre Dumais

**Affiliations:** 1 University of Montreal; 2 Institute Philippe Pinel of Montreal

## Abstract

**Background:**

While many pharmacological and psychosocial interventions are available, many treatment-resistant schizophrenia patients continue to suffer from persistent psychotic symptoms, mainly auditory verbal hallucinations (AVH). Recently, a psychological therapy using computerized technology has shown large therapeutic effects on AVH severity by enabling patients to engage in a dialogue with a representation of their distressing voices. These very hopeful results have been extended by our team using immersive 3D virtual reality. The results of VR therapy (VRT) in our pilot trial involving 15 schizophrenia patients with refractory AVH were clinically promising for the severity and distress related to hallucinations, illness symptomatology, depressive symptoms and quality of life. Notably, clinical improvements of our pilot remained significant at our 3-month follow-up. Such findings suggest that VRT seems to be a highly promising intervention for refractory AVH.

**Methods:**

To further research in this field, the primary goal of this randomized-controlled trial is to show that VRT is superior to a widely utilized psychotherapy, that is Cognitive behavioral therapy (CBT), for the treatment of persistent auditory verbal hallucinations in schizophrenia. Our secondary goal is to examine the effects of these interventions on beliefs about voices, illness symptomatology, mood symptoms (anxiety and depression), self-esteem, level of functioning and quality of life.

This is a single-blinded randomized-controlled, single-site parallel study of VRT versus CBT. Each treatment group will include 52 randomized participants (assuming 20% attrition) of over 18 years of age hearing persecutory voices and suffering from treatment resistant schizophrenia or schizoaffective disorder. Diagnoses will be established with the Structured Interview for DSM-V. Patients will be included if they have been hearing persecutor voices that did not respond to ≥2 antipsychotic trials.

VRT comprises of 9 weekly sessions: 1 avatar creation session and 8 therapeutic sessions, where the patients are confronted to their reproduced hallucinatory experience and are encouraged to enter in a dialogue with their virtual persecutor. CBT includes 9 weekly sessions consisting of learning modules and task assignments. Subjects will be evaluated at baseline and post-treatment to assess primary (auditory verbal hallucinations as measured with the Psychotic Symptoms Rating Scale) and secondary outcomes. Mixed Anova analyses will be performed to measure and compare the effects of both interventions.

**Results:**

Presently, 37 patients have been recruited and 9 have abandoned the study. Our preliminary results on 28 patients show that there is no significant difference between the treatment conditions for all our measures. As expected, more participants will be required to show the superiority of VRT over CBT. However, when performing separate ANOVA analyses for each condition, VRT shows significant improvements of auditory verbal hallucinations severity after the treatment (on our primary outcome) contrarily to CBT. VRT also produced significant decreases on the beliefs that voices are omnipotent and malevolent, on psychotic symptomatology, depressive symptoms and an increase on quality of life. CBT obtained no significant improvements.

**Discussion:**

While limited by the small number of patients, such findings are nonetheless already supporting the hypothesis of the superiority of VRT on auditory verbal hallucinations. As expected, a moderate effect is found for our adapted short CBT for psychosis, though not significant at this point. The current trial will contribute to the validation of a novel innovative approach answering a fundamental clinical need.

